# Editorial: Loudness: From Neuroscience to Perception

**DOI:** 10.3389/fpsyg.2021.785093

**Published:** 2021-11-15

**Authors:** Sabine Meunier, Maaike Van Eeckhoutte, Brian C. J. Moore

**Affiliations:** ^1^Aix Marseille Univ, CNRS, Centrale Marseille, LMA, Marseille, France; ^2^Hearing Systems, Department of Health Technology, Technical University of Denmark, Lyngby, Denmark; ^3^Copenhagen Hearing and Balance Center, Ear, Nose, and Throat (ENT) and Audiology Clinic, Rigshospitalet, Copenhagen University Hospital, Copenhagen, Denmark; ^4^Cambridge Hearing Group, Department of Psychology, University of Cambridge, Cambridge, United Kingdom

**Keywords:** loudness, psychophysics, temporal and frequency integration, binaural loudness, hearing aids and cochlear implant

Loudness is the sensation that allows judgment of whether a sound is strong or soft. Sounds can be characterized by several perceptual features and among them loudness plays an important role. Loudness is very important for sound quality. Noise annoyance is mainly influenced by loudness, because, in most situations, the louder the sound, the more annoying it is. It is very important to control loudness for users of hearing aids and cochlear implants, for whom the loudness of sounds must be appropriate and the temporal fluctuations in loudness (particularly for speech) must be well-reproduced. Understanding how the percept of loudness is formed in the auditory system and how it is coded is therefore of great importance.

This special issue includes nine articles on loudness, mainly using psychoacoustical approaches, and ranging from theoretical issues to clinical applications. The issue explores psychophysics, loudness measurement, multisensory integration, the influence of the temporal and frequency characteristics of sounds on loudness, the way that loudness is combined across the two ears, and clinical applications to hearing aids and cochlear implants.

The article by Zeng presents a unified theory of psychophysical laws in auditory intensity perception. There has been a long history of psychophysical laws that attempt to relate the physical sound intensity of a stimulus to its perceived magnitude or loudness. The first approach was published by Fechner in 1860, who used just noticeable differences to infer that loudness is a logarithmic function of sound intensity. Over the years, Fechner's original assumption has been criticized and modified and a widely accepted view is that loudness is a compressive power function of sound intensity; this relationship is sometimes called Steven's power law. In this paper, Zeng reviews previous theories based on just noticeable differences and integrates them in a new unified theory, thereby also showing the validity of Fechner's original idea for a range of hearing situations.

The measurement of loudness is discussed in two articles. The article by Fultz et al. deals with categorical loudness scaling, a procedure that is often used for measuring the growth of loudness with increasing stimulus intensity. Some authors have proposed that categorical loudness scaling should be used in the fitting of hearing aids, but this requires time-efficient tests. Aiming to make categorical loudness scaling more efficient, this article describes a comparison of a “traditional” method using fixed stimulus levels with a method using Bayesian inference to select stimulus parameters that yield the maximum expected information gain during data collection. The article discusses methods for decreasing the test time, while maintaining test-retest reliability and accuracy, and it further discusses optimizations. In their study on the moment-by-moment loudness assessment of time-varying sounds, Schlittenlacher and Ellermeier used continuous cross-modality matching between line length and loudness (and vice versa) for musical excerpts of either rock or classical music. They found that line length is highly correlated with long-term loudness calculated using the time-varying loudness (TVL) model of loudness (Moore et al., 2018), showing the reliability of the method. Their results provide some support for the time constant (temporal portions of the sound that affect momentary judgment) of 750 ms used in the TVL model. As expected, because of the regression effect, the line-length adjustment task yielded an exponent of the loudness function smaller than predicted by Steven's power law.

The article by Fischenich et al. explores spectro-temporal processes that influence loudness. Using the correlations between loudness judgments and the levels of brief temporal segments within longer sounds, they show that temporal weights in loudness judgments are frequency specific. This result suggests that temporal integration precedes spectral integration. This is consistent with the most recent version of the TVL model of loudness (Moore et al., 2016, 2018) and with recent neurophysiological data (Thwaites et al., 2017).

Two papers deal with the way that loudness combines across ears, often referred to as binaural loudness. In one paper, Denk et al. explore the “missing 6 dB” (the often-reported but sometimes disputed claim that a sound presented *via* headphones needs to have a 6 dB higher level at the eardrum than the same sound presented *via* a loudspeaker in order to elicit the same loudness). In a task where the listener adjusted the level of the sound presented *via* headphones to match the loudness of the same sound presented *via* a loudspeaker, they found that this mismatch was large at low frequencies but largely disappeared at high frequencies. The mismatch decreased when the interaural coherence (a measure of the correlation of the sound across the two ears) decreased, i.e., when the sound appeared to be more diffuse. Surprisingly, the mismatch was different in two different anechoic rooms whereas there was no difference between two non-anechoic rooms. Thus, the different results found in the literature concerning the “missing 6 dB” may be related to differences in the experimental conditions (reproduction mode, room, stimuli). The paper of Pieper et al. is concerned with Individualized Loudness Models (ILMs), which might help in the fitting of hearing aids in order to improve audibility, comfort and naturalness. Loudness models applied to impaired hearing take into account individual frequency-dependent reductions of cochlear gain and compression produced by hearing loss. Pieper et al. argue that, in addition, ILMs should take into account individual differences in binaural loudness summation. They propose an extension of a monaural loudness model “toward an individual binaural loudness model for hearing aid fitting and development.”

The paper by McKay describes the application of three loudness models to the perception of loudness by people with cochlear implants. One model is applied in the simple case of electrical stimuli applied to a single electrode. In this model, cochlear neural excitation is integrated over time using a central temporal integration window similar to that used in models of loudness for normal hearing, such as the TVL model. The other more complex model (the “Detailed” model) is applied when multiple electrodes are stimulated within a short time interval. This model includes the effects of interaction between different electrodes. McKay also presents a “Practical” model, which is a simplified version of the “Detailed” model, and which can be used to predict the loudness of pulsatile electrical stimuli applied to multiple electrodes. The models have been applied to the development of novel signal processing strategies that aim to provide users of cochlear implants with a more natural perception of loudness.

In the paper by Sun et al., the authors use both behavioral experiments and electro-encephalography (EEG) to measure subtle multi-modal effects in loudness perception. Specifically, in four behavioral and EEG experiments, the authors show that visual-motor information from manual gestures modulates the loudness perception of consecutive sounds whose intensity changes, as well as the early auditory neural responses that correspond to the changes in loudness perception.

The paper by Berthomieu et al. describes mounting evidence that the loudness of sounds is influenced not only by their physical characteristics at the eardrum (intensity, spectrum temporal pattern, and binaural differences) but also by the manner of presentation, for example whether or not the sound source is visible, whether the sounds are presented *via* headphones or loudspeakers, or from “live” sources, such as a person talking, and whether or not the sounds are meaningful. Berthomieu et al. argue that loudness appears to depend on how listeners interpret the sound sources, notably whether they focus on the sound that reaches their ears (the proximal stimulus) or the sound as produced by the source (the distal stimulus). This distinction was made many years ago by Helmholtz who stated “…we are exceedingly well-trained in finding out by our sensations the objective nature of the objects around us, but we are completely unskilled in observing these sensations *per se*” [quoted in Warren (1981)]. Berthomieu et al. argue that whether the listener focusses on the proximal or distal stimulus depends on the instruction to the listener and on how the sound is interpreted. Many experiments on loudness perception have been set up so as to promote listening to the proximal stimulus, whereas in everyday life loudness may be more related to the distal stimulus.

## Author Contributions

All authors listed have made a substantial, direct and intellectual contribution to the work, and approved it for publication.

## Funding

BM was supported by the Engineering and Physical Sciences Research Council (UK, Grant Number RG78536).

## Conflict of Interest

The authors declare that the research was conducted in the absence of any commercial or financial relationships that could be construed as a potential conflict of interest.

## Publisher's Note

All claims expressed in this article are solely those of the authors and do not necessarily represent those of their affiliated organizations, or those of the publisher, the editors and the reviewers. Any product that may be evaluated in this article, or claim that may be made by its manufacturer, is not guaranteed or endorsed by the publisher.
